# 

**Published:** 1889-07-27

**Authors:** 


SATURDAY, JULY 27th, 1889.

**Two Famous Doctors** - 255
**A Protest against Pasteurism** - 256
**Turning over New Leaves.**—Songs of the Spindle - 256
**The Doctor's Pulpit.**—In Nature's School - 257
**The Story of the Insane from Year to Year.**—Asylum Reports - 258
**Florence Nightingale** - 259
**Notes and News** - 262
**Everybody's Page** - 264
**Annotations.** — Weariness—"A Reasonably-dressed Woman!"—Would-be Suicides - 265
**Sea-Sickness** - 266
**Her Heart's Mistake.** By E. D. Cumming. - 267
**Editor's Letter-Box.**—The Doctor's Critic Examined - 269
**Daises and Buttercups** - 269
**Medical Courtship** - 270
**Amusements and Relaxation** - 270
**Scraps and Gleanings** - 270

EXTRA SUPPLEMENT—THE NURSING MIRROR.

**En Passant.**—A Warning to Midwives—The Surgeon or Lady Superintendent—Sick Nurses at Addenbrookes—The Home at Felixstowe—Darenth Asylum—District Work at Brighton—The Hague Deaconesses - lxiii
**The British Nurses' Association** - lxiv
**Physiology for Nurses.**—No. 3. Baths and their Effects - lxv
**The Princess of Wales and the National Pension Fund** - lxvi
**Everybody's Opinion.**—The Pension Fund—Registration - lxvi
**Presentation** - lxvi
**Appointments—Vacancies** - lxvi
**Notes and Queries** - lxvi

